# Consensus on pediatric epilepsy surgery for young children: an investigation by the China Association Against Epilepsy task force on epilepsy surgery

**DOI:** 10.1186/s42494-023-00130-7

**Published:** 2023-08-14

**Authors:** Lixin Cai, Kai Zhang, Wenjing Zhou, Xiaoqiu Shao, Yuguang Guan, Tao Yu, Ye Wu, Shuhua Chen, Rui Zhao, Shuli Liang, Xun Wu, Guoming Luan, Yuwu Jiang, Jianguo Zhang, Xiaoyan Liu

**Affiliations:** 1 Pediatric Epilepsy Center, Peking University First Hospital, Beijing, 100000 China; 2 Department of Neurosurgery, Beijing Tiantan Hospital, Capital Medical University, Beijing, 100000 China; 3 Department of Epilepsy Center, Tsinghua University Yuquan Hospital, Beijing, 100000 China; 4 Department of Neurology, Beijing Tiantan Hospital, Capital Medical University, Beijing, 100000 China; 5 Department of Neurosurgery, Sanbo Brain Hospital, Capital Medical University, Beijing, 100000 China; 6 Department of Functional Neurosurgery, Xuanwu Hospital, Capital Medical University, Beijing, 100000 China; 7 Department of Pediatrics, Peking University First Hospital, Beijing, 100000 China; 8 Department of Neurology, Beijing Children’s Hospital, Capital Medical University, 100000 Beijing, China; 9 Department of Neurosurgery, Children’s Hospital of Fudan University, Shanghai, 200000 China; 10 Department of Functional Neurosurgery, Beijing Children’s Hospital, Capital Medical University, Beijing, 100000 China; 11 Department of Neurology, Peking University First Hospital, Beijing, 100000 China

**Keywords:** Epilepsy surgery, Young children, Consensus

## Abstract

Researchers have widely acknowledged the therapeutic value of epilepsy surgery for drug-resistant epilepsy. Nonetheless, there is a substantial gap in the surgical treatment for appropriate candidates owing to several factors, particularly in the population of young children. To standardize the protocols of preoperative evaluation and surgery of young children for epilepsy surgery, the China Association Against Epilepsy has appointed an expert task force to standardize the protocols of preoperative evaluation and surgery in pediatric epilepsy patients. It adopted the modified Delphi method and performed two rounds of surveys through an anonymous inquiry among 75 experts from four subgroups including pediatric neurologists, epileptologists, pediatric epilepsy surgeons, and functional neurosurgeons. The survey contents contained: (1) the participants, comprising children aged ≤ 6 years; (2) adopted DRE definition proposed by the International League Against Epilepsy in 2010; and (3) investigated epilepsy surgery, principally referring to curative epilepsy surgeries. The neuromodulation therapies were excluded because of the differences in treatment mechanisms from the above-mentioned surgeries. According to the Delphi process, a consensus was achieved for most aspects by incorporating two rounds of surveys including preoperative assessment, surgical strategies and techniques, and perioperative and long-term postoperative management, despite controversial opinions on certain items. We hope the results of this consensus will improve the level of surgical treatment and management of intractable epilepsy in young children.

## Background

 The incidence of epilepsy greatly varies in different age groups. According to the International League Against Epilepsy (ILAE), the incidence is the highest in the population aged < 5 years and > 65 years (> 60 per 100,000). Furthermore, the incidence of epilepsy in children aged < 1 year (82.1–118 per 100,000 person-years) surpasses that in older children (46 per 100,000 person-years) [1]. Based on a 30-year cohort study on anti-seizure medicine (ASM), initial ASM enabled 50.5% of the patients to remain seizure-free; the possibility of seizure freedom with the second ASM was 11.6%, with only an additional 4.1% seizure freedom following the third regimen [2]. Additionally, a single-center randomized trial of children aged ≤ 18 years with drug-resistant epilepsy (DRE) verified the advantage of epilepsy surgery over ASM in children with DRE [2]. Though the therapeutic value of epilepsy surgery for DRE has been widely acknowledged by researchers; nonetheless, there is a substantial gap in the surgical treatment for appropriate candidates owing to various factors [3].

Pediatric epilepsy surgery, particularly for children aged < 6 years, differs significantly from that for older children and adults in terms of the preoperative evaluation and surgical techniques. Most previous studies involved adult and pediatric cases; however, they had a limited sample size and poor homogeneity of the investigated population and approaches. Furthermore, clinical studies on pediatric epilepsy surgery typically focused on the surgical efficacy and prognosis, but seldom discussed preoperative evaluation and detailed surgical strategies/techniques. In addition, researchers have updated some of the previous concepts, procedures, and conclusions with the rapid development of preoperative evaluation and surgical techniques.

To further standardize the protocols of preoperative evaluation and pediatric epilepsy surgery, the China Association Against Epilepsy (CAAE) has appointed an expert task force (TF), with the aim of reaching a consensus based on extensive investigations and for actively promoting the development of pediatric epilepsy research.

## Methods

A consensus expert TF of pediatric epilepsy surgery was established for young children, which was composed of members with expertise in preoperative evaluation and surgical treatment. The participants were divided into four subgroups: (1) pediatric neurologists, (2) epileptologists, (3) pediatric epilepsy surgeons, and (4) functional neurosurgeons.

Survey contents included (1) the participants, comprising children aged ≤ 6 years; (2) adpoted DRE definition proposed by the ILAE in 2010 [4]; and (3) investigated epilepsy surgery, principally referring to curative epilepsy surgeries. For instance, the resection and disconnection of the epileptogenic zones, stereoelectrography (SEEG)- guided thermocoagulation, or magnetic resonance imaging (MRI)-guided laser interstitial thermotherapy (LITT). We excluded neuromodulation therapies.

We extracted studies on epilepsy surgery, epilepsy etiology, neuroimaging, children’s electroencephalogram (EEG), and children’s brain development, particularly those published within the past decade. Moreover, we retrieved guidelines or consensus on the ILAE website from a professional database (i.e., PubMed, Chinese National Knowledge Infrastructure, or Wan Fang database).

**Fig. 1:**
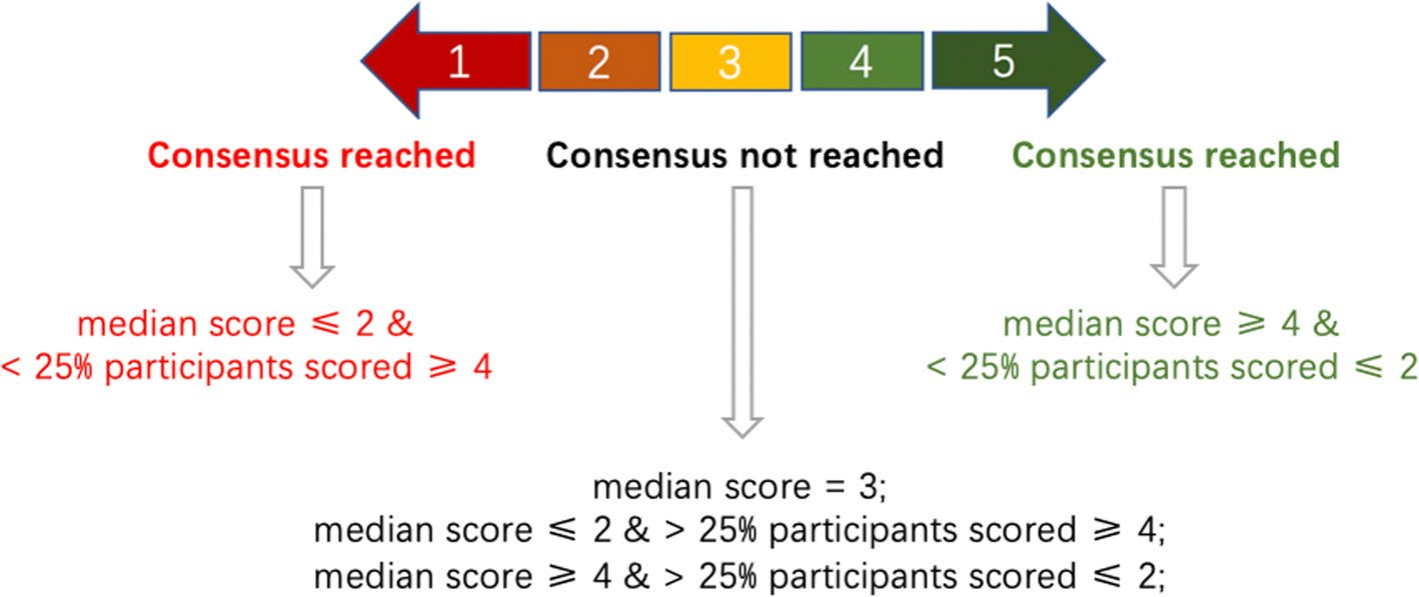
Consensus standards based on the modified Delphi method

Investigation Method: We applied the modified Delphi method to conduct two rounds of surveys through an anonymous inquiry. Based on the literature review, we extracted statements addressing problems related to pediatric epilepsy surgery for young children along with explanations through the first round of the survey. The participants were required to reply to the degree of approval for each view and offer their own proposals or suggestions for revision. Subsequently, issues that did not reach an agreement in the first round of the survey were listed without explanations such that the participants could independently grade the levels of recommendations in the second round. The survey responses were graded on 5-point Likert scale (Fig. 1). Owing to the different subspecialties involved, participants could grade issues that did not meet their expertise with “0” points (not involved in scoring statistics, but only in calculating the response rates). We considered the second score as the final result for inconsistent responses to similar issues from identical participants between the two surveys.

Consensus standards and statistical analysis: Consensus standards were based on the results of the retrieved Likert scale (Fig. 1). We used IBM SPSS (version 22.0) for statistical analysis. Measurement data are presented as the median (25 percentile, 75 percentile), and enumeration data are presented as percentages. We performed Pearson’s chi-square or continuous correction chi-square test to compare rates. Statistical significance was set at *P* < 0.05.

## Results

The first round of the questionnaire comprised 99 descriptive items and opinions on the age, preoperative evaluation, surgical strategy, perioperative management, and long-term postoperative management. Furthermore, 65 questions were designed in the second round, which aimed at a more specific description of particular questions or controversial issues from the first round of the survey. The feedback rate of the first questionnaire was 98.8% (85/86), and the response rate of each question was 68.7–85.9%. The feedback rate of the second questionnaire was 88.2% (75/85), with response rates of 55.4–83.1%. Among the 75 experts who completed both surveys, 27 (36%) were pediatric neurologists, 13 (17%) were epileptologists, 11 (15%) were pediatric epilepsy surgeons, and 24 (32%) were functional neurosurgeons. In addition, questions in neuroimaging, nuclear medicine, and pediatric development were designed with the help of experts in the aforementioned fields.

According to the Delphi process, a consensus was achieved for most aspects by incorporating two rounds of surveys, despite controversial opinions on certain items (emphasized in italics).

### Age groups for pediatric epilepsy surgery in young children

Postnatal development of the brain, characteristics of etiology, neuroimaging, EEG, seizure symptomatology, and other aspects in young pediatric patients with epilepsy are related to age [3–6]. The treatment modality is also significantly different from that of adult cases in terms of surgical strategies, surgical risks, and postoperative brain functional plasticity. Hence, it is highly recommended to refine the age groups further for pediatric epilepsy surgery in young children (Table 1) to formulate more precise and reasonable preoperative evaluation and surgical plans.

**Table 1:** Age groups for the pediatric epilepsy surgery in young children and key points

Age groups	Key points for pediatric epilepsy surgery (no comparison with older children and adults)
Early infancy (0–3 months)	Surgical risks, neuroimaging features, EEG features, and surgical strategies/techniques
Toddlerhood (< 3 years)	Neuroimaging features, EEG features, feasibility of SEEG, and surgical strategies/techniques
Preschool childhood (< 6 years)	EEG features and surgical strategies

*SEEG *Stereoelectrography, *EEG *Electroencephalogram

### Preoperative evaluation

All children with DRE should be referred to epilepsy centers for a comprehensive diagnosis and treatment evaluation [7]. The purposes of preoperative evaluation are as follows: (1) to confirm the diagnosis and classification of epilepsy, (2) to cofirm the etiology and surgical candidacy, (3) to localize the epileptogenic zone and perform surgical planning, and (4) to evaluate the benefits and risks of surgery.

#### Etiologic diagnosis

Compared to the etiologies of adult DRE, those of pediatric DRE are more complex and diverse. According to the ILAE 2017 epilepsy classification, [8] each etiology (structural, genetic, infectious, metabolic, immune, and unknown causes) should be systematically re-evaluated during the preoperative evaluation for selecting appropriate surgical candidates. First, clinicians should perform comprehensive medical history taking, physical examination, MRI, and necessary laboratory examinations to exclude genetic, metabolic, and degenerative diseases, autoimmune encephalitis, extensive brain injury, and other causes that are unsuitable for epilepsy surgery.

Genetic or genetic–structural disorders are the most common causes of DRE in young children with epilepsy. Genetic testing is recommended for the following conditions: DRE with an unknown etiology, family history of epilepsy, developmental epileptic encephalopathy, febrile seizure, MRI indicative of various malformations of cortical development (MCD), neurocutaneous syndrome, multiple apparent deformities or multiple system deformities, suspected genetic metabolic diseases, or neurodegenerative diseases. Moreover, genetic tests including Trio whole-exome sequencing and copy number variations are recommended. If necessary, Sanger sequencing or chromosome karyotype analysis is recommended [9].

#### Neuroimaging

##### MRI

MRI should be performed for all children with DRE. The TF recommends using the harmonized neuroimaging of epileptic structural sequences (HARNESS)-MRI with isotropic, millimetric three-dimensional T1 and fluid-attenuated inversion recovery (FLAIR) images.^11^ However, it is recommended to change high-resolution two-dimensional sub-millimetric T2-weighted images from coronal to axial planes to better identify MCD in infants. T2-FLAIR imaging is not necessary for neonatal MRI [10].

The myelination process in infants and young children continues after birth until 2 years of age; thus, MRI should be completed every 6–12 months for children with an unknown DRE etiology and/or unclear neuroimaging lesions until 36 months to identify potential brain structural abnormalities. The time gap between the latest MRI examination and preoperative evaluation should not exceed 6 months for patients aged < 3 years, and 1 year for those aged > 3 years [10–12]. Other special MRI sequences are not recommended as a routine examination item and are only recommended for certain pathologies or functional assessments (Table 2).

**Table 2:** The application of special MRI sequences

Special MRI sequences	Recommended application scope and purpose
Contrast enhanced MRI	Sturge–Weber syndrome, brain tumors, and vascular malformation; to determine the pathological diagnosis and extension of lesions.
Diffusion tensor imaging	Unilateral lesions involving motor cortex or the whole hemisphere; to evaluate the distribution of the corticospinal tract and motor function compensability.
DWI	During the sequelae stage of encephalitis, reviewing the DWI sequence in the acute stage of encephalitis is helpful for evaluating the extent of injury and potential epileptogenic zone in children.
Magnetic sensitivity-weighted imaging	Cavernous hemangioma and Sturge–Weber syndrome; to display tiny intracerebral hemorrhage.

*MRI *Magnetic resonance imaging, *DWI *Diffusion-weighted imaging

##### Positron emission computed tomography (PET)

18F-fluorodeoxyglucose (18F-FDG) PET is a routine examination for the preoperative evaluation of pediatric epilepsy. PET-computed tomography (CT) images have low tissue resolution; therefore, PET and MRI co-registration (post-processing) should be routinely performed to improve the resolution of localization. The interval between PET and MRI acquisition for co-registration should not exceed 3 months [13]. Frequent interictal discharges or seizures during the examination may compromise the interpretation of PET results. We should carefully inquire about the seizure condition during PET examination and refer to the recent EEG results [14].

*This investigation failed to reach a consensus on the application of PET-MRI (not the PET-MRI co-registration technique).* PET-MRI and PET-CT are equivalent in localizing the epileptogenic zone [15]. However, the MRI part has low resolution in most PET-MRI applications. Furthermore, the sequence of MRI in PET-MRI is incomplete, which can neither meet the requirements of HARNESS nor provide other useful information on CT. Therefore, the TF does not recommend PET-MRI as routine examination.

##### CT

CT scans of the brain should be available for tuberous sclerosis (TSC), focal cortical dysplasia (FCD), brain tumors, Sturge–Weber syndrome, cerebral vascular malformations, and other lesions before surgery. It is primarily used to detect focal calcifications.

##### Single photon emission computed tomography (SPECT)

 The seizure timing, detection environment, and injection time limits the subtraction of ictal-interictal SPECT co-registered to MRI, thus making its utilization difficult in young children. Therefore, this investigation failed to reach a consensus on the use of this technique for children with epilepsy aged < 6 years.

##### Functional MRI (fMRI)

 This investigation failed to reach a consensus on the application of fMRI to evaluate important functional areas in young children [16].

#### Neurophysiological examination

##### Long-term video electroencephalography (VEEG) monitoring

Long-term VEEG monitoring is routinely performed for preoperative evaluations. Regardless of the age, VEEG monitoring for preoperative evaluation should meet the technical requirements of the CAAE “Technical Guidelines for Clinical EEG”. We do not recommend the use of needle electrodes as sphenoid electrodes in children. Furthermore, the TF has emphasized the importance of multiple groups of surface electromyography recordings in determining the type of seizure in young children. Typically, at least three to five habitual seizures should be recorded. Young children often present with different seizure types, and each seizure type at the current stage should be recorded as much as possible. Semiology and EEG features in pediatric epilepsy may change with the age and development; therefore, all previous EEG records should be reviewed systematically. In case the interval between the presurgical evaluation and surgery exceeds 6 months or the semiology of seizure changes, previous evaluation conclusions should be modifed after reexamination of long-term VEEG [17].

##### Magnetoencephalogram (MEG)

*This investigation failed to reach a consensus on the application of MEG in young children.* Limited resources and cost–benefit ratios were the primary concerns.

##### Intracranial EEG (iEEG)

Currently, SEEG is the most commonly used invasive iEEG monitoring method in China. SEEG can be considered in the following situations for young children: (1) inconsistent results of noninvasive examinations, (2) the epileptogenic zone is adjacent to or overlaps the eloquent cortex, (3) an unclear boundary of epileptogenic lesions necessitating further confirmation of the epileptogenic network and resection area, and (4) planned SEEG-guided radiofrequency thermocoagulation (RF-TC) of the local epileptogenic lesion [18, 19].

 For negative MRI and PET finidings in infants aged < 3 years, the investigation failed to reach a consensus on the use of iEEG to localize the epileptogenic zone according to electroclinical clues [20].

*The TF failed to reach a consensus on the use of subdural electrodes to localize motor areas in children aged 3–6 years (median = 3 years). Overall, 40% and 52% of the two epilepsy surgeon groups did not recommend the use of subdural electrodes (≤ 2 points), which was significantly different from the pediatric and epileptologist groups (12% and 27%) (P = 0.027).* Following the panel discussion, the opinion of the epilepsy surgeon groups was prioritized on this issue.

##### Electrical cortical stimulation (ECS)

During SEEG recording in children, clinicians should routinely perform ECS to localize the functional areas and epileptogenic zones [21]. Stimulation in children often requires a higher current intensity than that in adults.

##### Wada test and awake anesthesia

The Wada test or awake anesthesia is not recommended for children aged < 6 years.

#### Neurodevelopmental assessment

First, clinicians should assess the gross motor skills, fine motor skills, language comprehension and expression ability, and handedness using physical examinations. Currently, there is no unified scheme for a developmental assessment in pediatric epilepsy surgery [22–24]. Table 3 summarizes the preoperative development assessment tests recommended by this consensus for pediatric patients with epilepsy. A development quotient (DQ) or intelligence quotient test should be performed in all children within 3 months before surgery, which should be re-evaluated for a prolonged interval.

**Table 3:** Recommended tests for the preoperative development assessment of pediatric patients with epilepsy

Assessment items	Recommended assessment scale	Applicable age
Developmental assessment	Griffiths Mental Development Scales	0–8 years
Intelligence assessment	Wechsler Preschool and Primary Scale of Intelligence-fourth edition	2 years 6 months–6 years 11 months
Adaptive behavior assessment	Adaptive Behavior Assessment System (children’s edition)	0–16 years
Motor	Peabody Developmental Motor Scale-Second Edition^a^	0–72 months
Language	Peabody Picture Vocabulary Test^a^	3 years 6 months–8 years
Spatial cognition	Visual-motor Integration Test^a^	≥ 2 years
Behavior	Child Behavior Checklist^a^	4–16 years
	The Conners’ Parent Rating Scales (attention-deficit/hyperactivity disorder)^a^	3–17 years
	ABC Parent Rating Scales (Autism Behavior Assessment Scale)^a^	< 6 years

^a^Other assessment scales can be added (but not limited to) according to the children’s development, behavior, and possible surgical area

#### Indications for epilepsy surgery

Typically, epileptogenic structural abnormalities observed on MRI in young children with DRE are important prerequisites for the surgical treatment. Currently, there is no clear definition of the indications for epilepsy surgery. This investigation focused on the characteristics of epilepsy surgery in young children and reached a consensus on most issues regarding the indications in the inquiry.

##### MRI and PET

Surgical treatment is not indicated for young children with an unknown etiology, negative MRI findings, or bilateral diffuse brain abnormalities regardless of seizure types. It is difficult to localize the epileptogenic zone and determine the resection regions in infants and young children based solely on electroclinical features. Regardless of positive or negative findings on MRI, PET displaying extensive or multifocal metabolic abnormalities that cannot be explained by the anatomo–electro–clinical features may indicate other potential causes or influencing factors. Under such circumstances, clinicians should carefully evaluate the surgical indications [25, 26].

##### Genetic factors related to MCD

Numerous MCDs are related to genetic factors, such as gene mutations, most of which are somatic mutations. Currently, somatic mutations can not be directly detected or verified before surgery. In some cases, MRI characteristics can provide some clues. For germline mutations of MCD-related genes (such as the pathogenic variation of mTOR, GATOR1 complex, and other genes detected in the peripheral blood), the TF recommends surgery for positive MRI findings and consistent anatomo-electro-clinical features. The influence of germline gene variations on surgical outcomes is unclear [27–33]; however, surgical decisions should be made cautiously in patients with negative MRI.

##### Non-MCD-related gene mutations

Gene mutations related to the ion channels, gene transcription and expression, and synaptic transmission can manifest as clinical childhood DRE with focal seizures. MRI scans of these patients often lack specific focal abnormalities, and they are not suitable surgical candidates, generally. A minority of cases with visible epileptogenic MCD or hippocampal sclerosis (double pathology) can be carefully evaluated for the possibility of surgical treatment in case of consistent anatomo–electro–clinical features for the possible epileptogenic zone. However, surgery cannot eliminate seizures related to gene mutations or can aggravate seizures (such as SCN1A-related Dravet syndrome) [34–38].

##### Acquired diffuse or bilateral brain injury

In most cases, curative surgery is not indicated for certain etiologies, such as hypoxic ischemic encephalopathy, hypoglycemic brain injury, viral encephalitis, and autoimmune encephalitis. On combining these injuries with unilateral hippocampal sclerosis or other focal lesions (such as focal atrophy or encephalomalacia), clinicians can carefully evaluate the possibility of surgical treatment for focal epileptogenic lesions based on consistent anatomo–electro–clinical features. However, they should consider the possibility of a potential epileptogenic zone in other brain regions, which may affect the long-term surgical outcomes. Chronic epilepsy following autoimmune encephalitis is usually not indicated for surgical treatment [39, 40].

##### Brain structural abnormalities not related to epilepsy

Lesions, such as arachnoid cyst in the choroidal fissure and focal white matter demyelination, are not related to epilepsy and do not require any surgical treatment. A minority of arachnoid cysts with high tension may significantly displace the adjacent cortex. Under such circumstance, clinicians should carefully analyze if the adjacent cortex is epileptogenic.

##### Severe developmental retardation

For severe developmental retardation with a DQ of < 20, other potential causes related to epilepsy should be completely considered regardless of brain structural abnormalities. Surgical indications and outcomes (including seizure and developmental outcomes) should be carefully weighed before making surgical decisions [41].

#### Anatomo–electro–clinical principles

The basic principles and methods of analyzing the anatomo**–**electro**–**clinical correlation in adult epilepsy surgery are also applicable to children; however, clinicians should pay attention to the development-related characteristics of young children. The semiology of focal epilepsies may be atypical, which is not reliable for the localization of the epileptogenic zone. Further, EEG often demonstrates extensive or multifocal discharges, which makes it difficult to provide localizing information. Moreover, focal epileptogenic structural abnormality on MRI provides the most important evidence for preoperative evaluation in young epilepsy children. However, numerous factors (imaging resolution, brain development process, and the reviewer’s experience and focus) may influence positive MRI findings. The focal abnormal hypometabolism of interictal PET can provide important information for the lateralization and localization of the epileptogenic zone; nonetheless, the area of hypometabolism may be extensive and involve the seizure propagation network. Therefore, the extent of surgical resection cannot be based on the results of PET alone [42, 43].

A child with DRE can be considered for preoperative evaluation regardless of various seizure types and EEG patterns if MRI indicates focal epileptogenic lesions and the findings are essentially consistent with the hypometabolic distribution of PET. Surgery can be considered for children aged 3–6 years in case of negative MRI findings accompanied by focal metabolic abnormalities on PET, which is essentially consistent with the electroclinical lateralization and/or localization of the focal seizures. Intracranial EEG is usually needed to confirm the extension of surgical resection [44].

### Strategies and techniques for epilepsy surgery

#### General principles and requirements

The surgical strategies and methods of epilepsy surgery in young children differ from those in adults [45]. The neurosurgical team conducting pediatric epilepsy surgery should have the basic skills of pediatric neurosurgery and epilepsy surgery. Basic relevant equipments are essential, which include micro-neurosurgery equipment, surgical robots (for SEEG implantation), and intraoperative neurophysiological monitoring (IONM) instruments.

Body weight is a vital factor affecting the surgical safety in infants. The commonly accepted safe weight for surgery is > 10 kg [46]. The risk of surgery increases with the decrease in the body weight.

Considering the situation in China, this consensus does not propose mandatory standards for epilepsy centers that can conduct pediatric epilepsy surgeries [47]. However, the TF recommends that more difficult and complex pediatric epilepsy surgeries (such as hemispherotomy, multilobar resections or disconnection, or surgery involving the Rolandic area) should be performed in a tertiary epilepsy center with advanced technology and equipment to guarantee the safety and efficacy of surgical treatment. The anesthesiologist should be experienced in managing young children, and the epilepsy center should have a pediatric intensive or similar care unit.

#### Surgical timing

Numerous clinical studies have demonstrated that early surgery with satisfactory seizure control exerts a significant positive effect on improving the overall prognosis in children with DRE and clear surgical indications [48]. Some etiologies are susceptible to becoming DRE with an early seizure onset (hemimegalencephaly or FCD). In case of frequent daily seizures or status epilepticus refractory to ASMs, clinicians should consider the possibility of early surgical intervention [49].

In young children with structural epileptogenic lesions (excluding tumors) on MRI, surgical treatment may not be the first choice when they remain seizure-free for a prolonged duration. However, epilepsy surgery should be considered in cases of clinical recurrence, severe ASMs adverse effects, or cognitive decline caused by persistent epileptic discharge.

#### Surgical strategy

Surgical strategies for young children must be developed according to the etiology, location of epileptic focus, and extent of the lesion.

##### MCD

Resection or disconnection of the epileptogenic zone can be directly performed in young children with MCD (excluding TSC, polymicrogyria, and paraventricular gray matter heterotopia) and consistent anatomo–electro–clinical correlations. Some small epileptogenic lesions can also be treated with SEEG-guided thermocoagulation or LITT. When the lesion involves multiple lobes with a relatively limited epileptogenic region or when one lesion is accompanied by bilateral diffuse discharges, a planned, staged surgery may be considered, in which limited resection of the susceptible epileptogenic zone or callosotomy can be performed during first-stage surgery. Subsequently, the entire lesion is resected in the second-stage surgery if necessary [50, 51]. For staged surgery, clinicians should establish a prospective surgery plan and completely inform the family about the prognosis of the surgery and possibility of reoperation. However, a one-stage complete resection or disconnection should be performed for single-lobe lesions whenever possible [52].

##### TSC

Epileptogenic tubers can be directly resected in case of consistent anatomo–electro–clinical correlations during noninvasive preoperative assessment. However, there is no consensus on the role of SEEG in identifying the responsible tubers when it is difficult to identify epileptogenic tubers by scalp EEG and symptomatology [53].

##### Polymicrogyria and gray matter heterotopia

The structural lesions in polymicrogyria or gray matter heterotopia are not necessarily epileptogenic and may be functional. SEEG is often required to identify the epileptogenic and functional areas. Therefore, clinicians can perform restricted resection instead of the complete removal of structural abnormalities [54].

##### Long-term epilepsy-associated tumors (LEATs)

LEATs are relatively common in young children, with seizures as the only clinical symptom. *There was no consensus in this survey on the need for surgery in children with LEAT and well-controlled seizures under medical treatment.* Following discussion, the TF agreed on performing early surgery and clarifying the pathology for patients with LEAT regardless of a seizure attack.

##### Rasmussen encephalitis

Currently, hemispherotomy is the only effective treatment for intractable seizures in Rasmussen encephalitis; however, it is difficult to determine the timing of hemispherotomy following diagnosis. It is better to perform surgery before the age of 10 years for maximum recovery of postoperative neurological function [55, 56].

##### Sturge–Weber syndrome

The timing and extent of surgery should be comprehensively evaluated according to the age at seizure onset, frequency of seizures, occurrence of status epilepticus, areas of cortical involvement, and degree of motor impairment and cognitive regression. The extent of surgery should be designed according to the areas with leptomeningial enhancement. Most young children with Sturge–Weber undergo hemispherotomy or multilobar surgery [57].

##### Hippocampal sclerosis

In young children with DRE, hippocampal sclerosis is rare as an independent epileptic focus. Therefore, further investigation of the underlying causes and evaluation of epileptogenic networks are required. Epilepsy surgery with the simple removal of the atrophic hippocampus should be cautiously performed in young children [58].

##### Lesions with rolandic area involvement

For young children with frequent seizures, if the lesion involves the Rolandic area, it will inevitably lead to contralateral motor impairment in the natural course. Even if there is no obvious hemiparesis, clinicians should consider early surgery with complete resection of the epileptogenic lesion. Diffusion tensor imaging should be performed preoperatively to assess functional connectivity. In addition, IONM should be performed to protect motor function.

##### Lesions involved in the language area

The language function of patients aged < 6 years displays strong plasticity. Therefore, language lateralization may be ignored in presurgical evaluation even if the surgery involves the classical language cortex for children aged < 6 years, particularly in those with cognitive and language developmental problems [59].

### Surgical procedure

#### Hemispherotomy or multilobar disconnection

Disconnection surgeries, such as hemispherotomy, temporoparietal disconnection, and frontal lobe disconnection, are recommended for epileptogenic lesions involving hemispheres, multiple lobes, or unilobe. Compared to the anatomical resection, they have several advantages, including less bleeding, shorter operation time, fewer complications, and quicker postoperative recovery. Hemispherotomy should include insular disconnection; Otherwise, the seizure outcome will be compromised [60–62].

#### SEEG-guided RF-TC

Hypothalamic hamartoma, paraventricular nodular heterotopia, small FCD type IIB, and other focal epileptogenic lesions are the indications for SEEG-guided RF-TC. Furthermore, to achieve a better seizure outcomes, both the localization of the seizure onset zone and ablation volume by RF-TC should be considered during the planning of electrodes coverage [63, 64]. Large hypothalamic hamartoma may be treated with staged RF-TC. However, LITT should be the first choice in such cases.

#### MRI-guided LITT

LITT is indicated for children aged > 2 years demonstrating deep-seated small epileptogenic lesions, such as bottom-of-sulcus dysplasia, hypothalamic hamartoma, and heterotopia, which can possibly lead to good seizure outcomes [65].

#### SEEG and intraoperative monitoring

##### Application of SEEG in young children

Generally, SEEG should be performed only in children with a skull thickness of > 2 mm. The SEEG planning principles for adults can be applied to children. However, the indications cannot be directly inferred from those for adults as there are distinctive features in the epileptogenic lesions and epileptic networks. Invasive evaluation with SEEG or subdural electrodes is not recommended in children aged < 3 years if neither MRI nor PET indicates the presence of a distinct lesion [19, 66–69]. In patients with short seizure duration (epileptic spasm, myoclonus, and atonic seizures), if there is no obvious abnormality on MRI despite the findings of focal hypometabolism on PET images, it is difficult to raise a reasonable hypothesis of the epileptogenic network. MCD involving the unilobe, multilobes, or hemisphere in young children aged < 3 years usually requires complete resective or disconnection surgery of potentially epileptogenic lesions to achieve good seizure outcomes, despite the identification of regional or multifocal seizure onset within the MCD by SEEG. In conclusion, complete resection of dysplastic lesions on MRI results in a good prognosis, [70, 71] and the application of SEEG commonly does not alter the final surgical strategy [52].

##### Electrocorticogram (ECoG)

ECoG is valuable in determining the extent of resection [72].

##### IONM

In young children with a risk of intraoperative motor cortex or corticospinal tract injury, it is difficult to perform noninvasive functional assessments before surgery. Therefore, IONM is strongly recommended. Based on the results of somatosensory and motor evoked potentials, motor function can be effectively protected by monitoring the continuous compound muscle action potential.

### Pathological sampling and diagnosis

Pathological diagnosis is important in determining the etiology, pathogenesis, and seizure outcome. Regardless of resective or disconnective surgery, the epilepsy surgeon should standardize the sampling and inspection processes. Epileptogenic lesions in the brain should be obtained and sampled to the greatest possible extent. In case of a large lesion (such as in multilobar surgery), multiple samples should be collected in discrete regions of the brain. It is essential to enhance the collaboration among neuroradiologists, genecitists, and pathologists for correct pathological diagnosis.

### Perioperative management

Perioperative management is important to guarantee the safety of surgery, reduce the incidence of postoperative complications, and shorten hospitalization. For children undergoing ketogenic diet or adrenocorticotropic hormone therapy, it is recommended to suspend these therapies before surgery. Generally, ASMs should be unchanged before surgery. Following viral encephalitis (e.g., herpes simplex encephalitis), the TF recommends routine perioperative antiviral therapy to prevent viral reactivation. The dosage of the recommended prophylactic acyclovir therapy is 10 mg/kg/dose every 8 h beginning 3 days prior to the surgery and continuing for 7–10 days after surgery [39].

An epidural drainage is recommended, but should not exceed 48 h postoperatively. A subdural drainage tube for < 1 week is recommended following hemispherectomy or hemispherotomy to prevent aseptic meningitis and long-term complications [61, 62]. Short-term treatment with steroids can be administered to prevent brain edema. The TF recommends performing CT within 24 h of surgery, focusing on postoperative hemorrhage and edema.

Postoperative complications extending the hospitalization in epilepsy surgery include aseptic fever, increased heart rate, and transient focal seizures caused by cerebral cortex edema. Long-term complications include subdural fluid accumulation, hydrocephalus, and epidural hematoma. Good surgical techniques are associated with fewer long-term surgical complications [73].

### Long-term postoperative management

#### Postoperative follow-up and further consultation

Prolonged ASM treatment and long-term follow-up (3–5 years) are required postoperatively. ASM should be adjusted according to seizure outcome. If the patient is receiving multiple ASMs before surgery, tapering of one or two ASMs can be initiated immediately after surgery. After 1 year of seizure freedom following surgery, ASM can be reduced gradually until the patient eventually withdraws the medication on reaching > 2 years of seizure freedom following surgery with normal or significantly improved EEG results. ASM should be resumed if seizures relapse during ASM discontinuation, which does not affect the seizure prognosis. However, patients with the failure of ASM discontinuation have a lower possibility of drug withdrawal in the future. Therefore, clinicians must cautiously attempt to completely withdraw ASM the second time [74].

Preoperative evaluation can be repeated during postoperative seizure recurrence to determine the causes of recurrence and necessity for reoperation if recurrent seizure is intractable to ASMs. Reoperation often requires more careful and cautious evaluation and larger size of resection (e.g., unilobar, multilobar, or hemisphere) [21]. The seizure-free rate following the third operation is low after a failed second operation, thereby indicating possible surgical resistant epilepsy [18, 75, 76].

#### Postoperative rehabilitation

Numerous children with intractable epilepsy require long-term rehabilitation in various aspects following surgery to achieve a good functional recovery. Parents should be instructed to conduct rehabilitation training in professional rehabilitation institutions for their children. Earlier rehabilitation leads to better functional recovery. Rehabilitation is recommended to persist for a relatively long period.

## Conclusions

Epilepsy surgery in young children is different from that in older children and adults in several respects. The TF adopted the Delphi survey method and conducted two rounds of anonymous interviews with 75 experts from four subgroups. Eventually, we reached a general consensus on most issues involving preoperative assessment, surgical strategies and techniques, and perioperative and long-term postoperative management. These findings will improve the level of surgical treatment and general management of intractable epilepsy in young children and promote multidisciplinary cooperation. On some issues that a consensus could not be reached, the TF provided suggestions following an in-depth discussion. The TF remained neutral on very few issues, including some workups that are difficult to manipulate in young children.

## Data Availability

Not applicable.

## Abbreviations

ASM: Anti-seizure medicine
CAAE: China Association Against Epilepsy
CT: Computed tomography
DRE: Drug-resistant epilepsy
DQ: Development quotient
ECoG: Electrocorticogram
ECS: Electrical cortical stimulation
EEG: Electroencephalogram
FCD: Focal cortical dysplasia
FLAIR: Fluid-attenuated inversion recovery
iEEG: Intracranial EEG
ILAE: International League Against Epilepsy
IONM: Intraoperative neurophysiological monitoring
LEATs: Long-term epilepsy-associated tumors
LITT: Laser interstitial thermotherapy
MCD: Malformations of cortical development
MEG: Magnetoencephalogram
MRI: Magnetic resonance imaging
PET: Positron emission computed tomography
RF-TC: Radiofrequency thermocoagulation
SEEG: Stereoelectrography
SPECT: Single photon emission computed tomography
TF: Task force
TSC: Tuberous sclerosis
VEEG: Video electroencephalography
